# Evolution of plasticity in the city: urban acorn ants can better tolerate more rapid increases in environmental temperature

**DOI:** 10.1093/conphys/coy030

**Published:** 2018-06-14

**Authors:** Sarah E Diamond, Lacy D Chick, Abe Perez, Stephanie A Strickler, Crystal Zhao

**Affiliations:** 1 Department of Biology, Case Western Reserve University, 2080 Adelbert Rd., Cleveland, OH, USA; 2 Hathaway Brown School, 19600 North Park Boulevard, Shaker Heights, OH, USA

**Keywords:** Thermal tolerance, microclimate, physiology, phenotypic plasticity, evolution, urban heat island

## Abstract

Because cities contain high levels of impervious surfaces and diminished buffering effects of vegetation cover, urbanized environments can warm faster over the day and exhibit more rapid warming over space due to greater thermal heterogeneity in these environments. Whether organismal physiologies can adapt to these more rapid spatio-temporal changes in temperature rise within cities is unknown, and exploring these responses can inform not only how plastic and evolutionary mechanisms shape organismal physiologies, but also the potential for organisms to cope with urban development. Here, we examined how plasticity in thermal tolerance under faster and slower rates of temperature change might evolve in response to the more rapid spatio-temporal temperature rise in cities. We focused on acorn ants, a temperature-sensitive, ground-dwelling ant species that makes its home inside hollowed out acorns. We reared acorn ant colonies from urban and rural populations under a common garden design in the laboratory and assessed the thermal tolerances of F1 offspring workers using both fast (1°C min^−1^) and slow (0.2°C min^−1^) rates of temperature change. Relative to the rural population, the urban population exhibited higher heat tolerance when the temperature was increased quickly, providing evidence that temperature ramp-rate plasticity evolved in the urban population. This result was correlated with both faster rates of diurnal warming in urban acorn ant nest sites and greater spatial heterogeneity in environmental temperature across urban foraging areas. By contrast, rates of diurnal cooling in acorn ant nest sites were similar across urban and rural habitats, and correspondingly, we found that urban and rural populations responded similarly to variation in the rate of temperature decrease when we assessed cold tolerance. Our study highlights the importance of considering not only evolutionary differentiation in trait means across urbanization gradients, but also how trait plasticity might or might not evolve.

## Teaser

Cities often warm at faster rates during the day and across space. The mechanisms underlying how organisms respond to these climatic changes in cities are largely unknown. This study finds evidence that the heat tolerance of an acorn-dwelling ant species has evolved rapidly to cope with these faster rates of urban warming.

## Introduction

Phenotypic changes in populations across rural-to-urban gradients are evident across a wide range of taxa (Alberti *et al.*, 2017), but the mechanisms governing these shifts in phenotype are not well understood. A key mechanistic question is whether urban-driven phenotypic shifts are entirely the result of plasticity, or whether there is a role for evolutionary change (Diamond and Martin, 2016; Merilä and Hendry, 2014). The argument for the dominance of plastic responses to cities seems plausible given the rapid timeframe over which urbanization processes typically occur, and the generally long timeframes over which evolution is thought to occur. Yet, there is more and more evidence for rapid evolution in cities (Johnson and Munshi-South, 2017). Disentangling the contributions of phenotypic plasticity and evolutionary change under urbanization is certainly an important research priority, as these mechanisms can occur over different timescales and operate under different constraints (Sgrò *et al.*, 2016) with sometimes divergent implications for vulnerability to global change (Franks *et al.*, 2014). But there is perhaps an underappreciated degree of subtlety here that can be overlooked in a framework that merely parses phenotypic variation into plastic and evolved components. And that subtlety has to do with the fact that plasticity itself can evolve (Ghalambor *et al.*, 2007).

Why might we expect plasticity to evolve in cities? One likely candidate for evolved plasticity lies in the physiological tolerance of temperature change, particularly as rates of temperature change over time and space differ substantially among urban and rural habitats. Although cities are often warmer on average (by several °C) compared with nearby rural habitats (Imhoff *et al.*, 2010), diurnal rates of temperature change, especially the rate of warming from nighttime to peak daytime temperatures, and the rate of warming across space are often more rapid in urban areas, owing to the diminished buffering effects of vegetation cover in developed areas (Adams and Smith, 2014). As a consequence, we might then expect urban populations to evolve a stronger plastic response in heat tolerance to rapid warming, i.e. that urban populations will achieve relatively higher heat tolerance when environmental temperature is increased quickly compared with rural populations. Shifts in estimated thermal tolerance as a function of the rate of temperature change (the “ramp rate”) are well documented for a number of ectothermic species (Allen *et al.*, 2016; Castañeda *et al.*, 2015; Chown *et al.*, 2009; Moyano *et al.*, 2017). However, the nature of this variation has generally been explored in context of the putative trade-offs between the ecological relevance of slower ramp rates versus the minimization of confounding acclimation effects not due to temperature stress (i.e. starvation and desiccation stress) associated with faster ramp rates (Terblanche *et al.*, 2011). Yet, in natural settings, organisms may experience slower and faster rates of temperature change, though few studies have quantified relevant rates of temperature change in the field (Woods *et al.*, 2015), and even fewer have examined the evolution of tolerance plasticity in response to different rates of environmental temperature change (but see Allen *et al.* (2016) for recent comparative work demonstrating interspecific variation in tolerance plasticity across different temperature ramp rates).

Here we use cities to explore how environmental variation in the rate of temperature change among urban and rural populations alters the plastic expression of thermal tolerance, and how this plasticity might evolve. Our study organism, the acorn ant (*Temnothorax curvispinosus*) is highly sensitive to temperature, including in development rate (Penick *et al.*, 2017), running speed (Maclean *et al.*, 2017) and thermal tolerance (Diamond *et al.*, 2013). Previously, we found evidence for evolved differences in heat and cold tolerance of acorn ants across an urbanization gradient such that urban populations exhibited increases in heat tolerance and losses in cold tolerance relative to rural populations (Diamond *et al.*, 2017); however, we did not explore whether plasticity had evolved, specifically whether urban acorn ants were able to better tolerate more rapid changes in environmental temperature. Like many ectothermic species, the thermal tolerances of ants appear to be sensitive to the rate of temperature change (Ribeiro *et al.*, 2012).

In this study, we quantified ecologically relevant rates of temperature change during the growing season for urban and rural acorn ants in the field, and linked these environmental differences with heat tolerance responses to faster and slower rates of temperature change in the laboratory. Through field-based recordings of environmental temperature and a laboratory common garden experiment, we evaluated the evidence for: (i) whether acorn ant nest sites warm more rapidly over time in urban habitats; (ii) whether the magnitude of spatial temperature change across the foraging area is greater in urban habitats; and (iii) whether differences in the rate of temperature change over time and space among urban and rural habitats are consistent with physiological responses to rate of temperature change. Specifically, we examined whether there has been evolutionary divergence in temperature ramp-rate plasticity such that urban populations of acorn ants are better able to tolerate more rapid increases in temperature change over space and time compared with rural populations. Although we focused on heat tolerance and spatio-temporal changes in environmental temperature during daytime hours when ants are most active within the growing season, we also examined plasticity in cold tolerance responses to different rates of temperature decrease and temporal changes in rates of cooling from peak daytime temperatures to nighttime within the growing season. The diurnal temperature change experienced by acorn ants over winter when ants are largely inactive and confined to the nest environment is likely the most relevant to link with ramp-rate plasticity in cold tolerance, though rates of cooling from day to night during the growing season may nonetheless provide a proxy. Although relevant physiological and environmental data are sparse, we hypothesized that urban populations might be less able to tolerate more rapid decreases in temperature compared with rural populations. This expectation is based on widespread signatures of nighttime-biased warming in many cities, which could lead to diminished rates of temperature decrease over diurnal timescales (Imhoff *et al.*, 2010). Alternatively, snow cover could homogenize rates of temperature decrease (Mitrus, 2013) that acorn ants experience in urban and rural habitats, potentially leading to a lack of population differentiation in cold tolerance plasticity across different rates of temperature decrease.

## Materials and methods

### Acorn ant collections

The acorn ant, *T. curvispinosus*, is widely distributed across the eastern United States, and commonly occurs in both urban and rural habitats. Colonies typically contain 50–200 workers and several queens at maturity. While queen lifespan can be quite high: from 5 up to 15 years (Keller, 1998), worker lifespan is comparatively short, on the order of one month (Herbers, 1989; Herbers and Johnson, 2007; Modlmeier *et al.*, 2013). The entire colony resides within pre-formed cavities in leaf litter, typically oak acorns, hickory nuts and small twigs; queens and immature workers are confined to the nest environment, while mature workers frequently leave the nest to forage on detritus (Herbers, 1989). As a consequence of their above-ground nesting habit, acorn ant colonies experience little buffering from changes in air temperature, compared with other species that diurnally and seasonally burrow underground to thermoregulate (Herbers and Johnson, 2007). Owing to their small body size, *T. curvispinosus* foraging distances are also quite small, often 1 m or less, and dispersal distances are similarly restricted, typically less than a few meters (Diamond *et al.*, 2012; Pratt, 2008). This small dispersal distance makes the species excellent for the study of urban thermal physiology, as highly structured populations can be found over small geographic scales (Stuart, 1987).

We collected acorn ant colonies from four urban and three rural sites across Cleveland, OH, USA (42°N latitude) in June 2016 (Supplementary Table 1). To identify urban and rural habitats, we used percent developed impervious surface area (ISA) (Imhoff *et al.*, 2010), such that urban sites were categorized as between 40 and 60% ISA, and rural sites were categorized as 0% ISA (note that ISA values were based on the 90 m radius around a particular site). Given that acorn ants are a cavity-nesting species and are reliant upon a supply of acorns for nest construction—acorn ants change their nest several times throughout the year (Alloway *et al.*, 1982; Herbers and Johnson, 2007)—our urban sites were effectively forested patches surrounded by developed areas. By contrast, rural sites were situated within large stands of continuous forest. Although the urbanization process may entail a variety of changes including to vegetation, habitat structure and the level of environmental contaminants, our comparison of forested patches within cities with rural forested areas allowed us to minimize the influence of these confounding variables while focusing on the temperature signal of urbanization.

### Thermal landscapes

We measured the temperature profiles in both acorn ant nest sites and the foraging areas surrounding individual nests. Because nests are static in space, we were most interested in the diurnal temperature changes in rural versus urban habitats for the nest environment. Within foraging areas, we were most interested in the spatial variation in temperature across the foraging area, i.e. how a foraging worker would experience temperature differences as they moved throughout the landscape.

**Figure 1:**
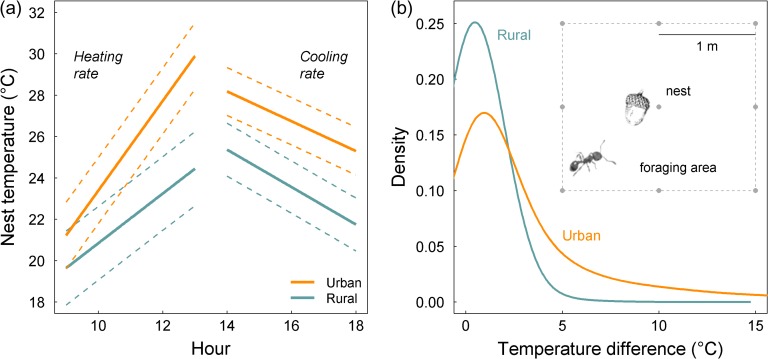
The rate of diurnal temperature change in urban and rural acorn ant nest sites and the magnitude of spatial temperature change across foraging areas. (**a**) Predicted values (mean and standard error) from models of rates of nest site warming (9 a.m. to 1 p.m.) and nest site cooling (2 p.m. to 6 p.m.) in urban and rural habitats during peak activity season (June–July). (**b**) Kernel density distributions (smoothed histograms) of the hourly pairwise differences (during the peak foraging interval, 9 a.m. to 1 p.m.) among foraging area temperatures for urban and rural habitats, again during peak activity season (June–July).

For June through July 2017 (this time of year captures peak activity season for acorn ants (Penick *et al.*, 2017)), we recorded temperature every 30 min from nine temperature loggers (iButtons, Maxim Integrated Thermochron data loggers DS1921G-F5) arrayed in a 2 m × 2 m square grid, each centered on a known acorn ant nest. We recorded from three rural grids and four urban grids (Fig. 1b). While some temperature loggers were situated in more shaded microhabitats and others were situated in light gaps, all temperature loggers were buffered against direct solar radiation by being encapsulated within 15 × 1.9 cm^2^ (l × w) sections of white PVC piping. The PVC piping was left open on either end to allow for air flow and laid flat against the ground to capture air temperature near the acorn ant nest and forager height. The central temperature logger within the grid and directly adjacent to the acorn ant nest was assayed for temporal changes in temperature, whereas the entire grid was used to assay spatial variation in temperature experienced by foraging ants. Although all temperature loggers were deployed in the same manner within each grid (i.e. housed within a PVC pipe shield), we only analyzed the central temperature logger next to the known nest site for temporal changes in temperature to best capture the microsite selection of nest locations by acorn ants. Because this approach limited our sample size for nest site temperature data, we added nest site temperature data recorded June–July in 2016 (using the same design of an iButton temperature logger housed within PVC piping) from one additional urban site and one additional rural site (the total number of nest site temperature recordings was: *n*_rural_ = 4, *n*_urban_ = 5).

The use of this standardized temperature logger design (iButtons housed within PVC piping) allowed us to make direct comparisons of environmental temperature across urban and rural habitats. Even if the temperature logger design does not capture precise nest and foraging area temperature (e.g. owing to differences in the size, shape and moisture levels of an acorn ant nest or how foraging ant body temperature responds to environmental temperature differences), it still captures the relative differences in spatio-temporal rates of environmental temperature change across urban and rural habitats.

### Common garden experiment

We collected urban and rural acorn ant colonies from the wild and reared them under common garden temperature treatments in the laboratory. After collection from the field, ant colonies were allowed to acclimate to laboratory conditions (~25°C, an intermediate temperature among our two temperature treatments) for ~24 h prior to being randomly assigned to two temperature treatments. Colonies from each source population (urban or rural) were reared under either a “warm” temperature regime mimicking peak urban foraging temperatures during the growing season (25°C nighttime temperature and 30°C daytime temperature) or a “cool” temperature regime mimicking peak rural foraging temperatures during the growing season (20°C nighttime temperature and 25°C daytime temperature) (see Diamond *et al.* (2017) for the development of rearing temperature regimes from environmental temperature data). Each treatment was kept under a standard long-day 14:10 L:D photoperiod, with the temperature change synced to the day–night cycle.

To generate F1 offspring for thermal tolerance testing, we reared colonies long-term, for a minimum of 5 weeks, in the laboratory common garden temperature treatments. The long acclimation period allowed sufficient time for turnover of workers within the colonies, i.e. the original workers died and the queens produced a new cohort of workers from eggs laid entirely within the common garden treatments, prior to assessment of thermal tolerance. This aspect of the experimental design allowed us to eliminate developmental acclimation effects from the field environment and some, but not all, potential maternal effects (Massamba-N’Siala *et al.*, 2014). Each colony was maintained individually in a 120 mL plastic cup. Resource tubes with sugar water (25% solution) and plain tap water were provided to colonies along with a continuous supply of dead mealworms.

At the end of the laboratory acclimation period, we used a dynamic temperature ramping protocol to assess the critical thermal maximum and minimum (CT_max_ and CT_min_), each defined as the loss of muscular coordination, which yields ecologically relevant metrics of heat and cold tolerance (Terblanche *et al.*, 2011). Ant workers were placed individually into 1.5 mL Eppendorf tubes with a cotton plug in the lid. Temperatures were manipulated using a dry block incubator (Boekel Scientific Tropicooler). We split the workers of a single colony into two groups for assessing thermal tolerance: in one group, the temperature was increased or decreased at a rate of 1°C min^−1^ (the “fast” ramp rate, within the context of our study) and in the other group, the temperature was increased or decreased at a rate of 0.2°C min^−1^ (the ‘slow’ ramp rate). Each tube was pulled out of the incubator once at the beginning of each temperature interval to check for muscular coordination of the individual ant, homogenizing the time spent inside and outside the incubator for each individual. Initial temperature for the estimation of CT_max_ was 34°C, and was 16°C for CT_min_. We assayed CT_max_ and CT_min_ using fast and slow rates of temperature change for a total of 30 colonies (number of colonies per population/temperature treatment/temperature rate: *n*_rural/cool/fast_ = 7; *n*_rural/warm/fast_ = 8; *n*_urban/cool/fast_ = 8; *n*_urban/warm/fast_ = 7; *n*_rural/cool/slow_ = 7; *n*_rural/warm/slow_ = 8; *n*_urban/cool/slow_ = 8; *n*_urban/warm/slow_ = 7). The mean number of workers assayed for each combination of CT_max_ and CT_min_, fast and slow rate of temperature change, warm and cool temperature treatment and urban and rural source population was 8.77 (± 2.40, 1 SD).

### Statistical analyses

All statistical analyses were performed in R (R Core Team, 2017). To examine how diurnal changes in nest site temperature profiles differed among urban and rural habitats, we constructed two linear mixed effects models using the *lmer* function from the {lme4} package (Bates *et al.*, 2015)—one model to assess nest site warming rates and one to assess nest site cooling rates. The nest site warming rate model included temperature as the response and hour (9 a.m. to 1 p.m.), source population (urban or rural) and their interaction. We included random intercept terms to account for autocorrelation due to the temperature logger and day from which temperatures were recorded. The nest site cooling rate model was constructed in a similar fashion to the nest site warming rate model except that the hours under consideration ranged from 2 p.m. to 6 p.m. The 9 a.m. to 1 p.m. and 2 p.m. to 6 p.m. time intervals represent the linear portions of the nest site warming and cooling periods over the course of a day (Supplementary Fig. S1). We did not model rates of temperature change between 1 p.m. and 2 p.m. owing to site- and day-level variation in the hour at which maximum daytime temperatures were reached. Sliding the time interval window by 1–2 h did not qualitatively alter our results (for models of both rates of temperature increase and decrease).

We also examined how spatial differences in foraging area temperature profiles differed among urban and rural habitats. Because we were specifically interested in the magnitude of temperature change across the spatial grid of the foraging area, we computed the Euclidean pairwise difference between each of the nine grid-arrayed temperature loggers for each hour within the peak foraging interval (9 a.m. to 1 p.m.) across the temperature monitoring period, and examined the distributions of these temperature profiles using kernel density estimation. We then constructed a simple linear model to assess whether the maximum of the urban and rural kernel density curves for the differences in temperature across the foraging area were significantly different from one another.

To examine how urban and rural populations differed in their ability to tolerate slow and fast rates of temperature increase (and decrease), we constructed linear mixed effects models separately for CT_max_ and CT_min_ as responses. As predictors, we included the main effects, two- and three-way interactions of the source population (urban or rural), rate of temperature change (fast or slow) and temperature treatment (warm or cool). We also included a random intercept for colony identity to account for non-independence among workers from the same colony. In the case of significant interactions among our predictor variables, we performed post-hoc analyses using the *lsmeans* function from the {lsmeans} package (Lenth, 2016).

Finally, to gain insight into potential genetic correlations among tolerance responses to different rates of temperature change, we examined the colony mean correlations between thermal tolerances assessed using the fast and slow rates of temperature change. We performed separate correlation analyses for each combination of source population, temperature treatment and tolerance metric (heat or cold tolerance). Owing to small sample sizes for colony mean tolerances, we performed Spearman’s rank correlation analysis.

## Results

### Ramp-rate plasticity in heat tolerance and rates of environmental temperature change

Rates of temperature increase, both over time at acorn ant nest sites and over space across foraging areas were greater in urban environments compared with rural environments (Fig. 1). The linear mixed model of nest site temperature as a function of hour during the diurnal period of nest site warming, source population, and their interaction revealed a statistically significant interaction between hour and source population (estimate and SE: 0.968 ± 0.166, *χ*^2^ = 34.1, *P* < 0.0001, *n*_obs_ = 1316, *n*_groups_ = 9 temperature loggers and 30 days), indicating the temperature rise per hour at the nest site was nearly a full degree Celsius greater in urban habitats compared with rural habitats. When we modeled the rate of temperature increase separately for each location, we found that the rate of temperature increase in the urban locations was 2.18 ± 0.130°C h^−1^ compared with 1.17 ± 0.0778°C h^−1^ in undeveloped forest habitat.

Likewise, we found that the magnitude of temperature change across the foraging area (estimated as the peak of the kernel density distribution of pairwise differences in environmental temperature among the temperature loggers within each foraging area grid) was greater in urban areas by nearly 0.5°C compared with rural areas (estimate and SE: 0.494 ± 0.110, *F* = 20.2, *P* = 0.00641, df = 5). We also computed the pairwise differences in environmental temperature among the temperature loggers within each foraging area while restricting comparisons to those temperature loggers exactly 1m away from one another (rather than all pairwise differences among the temperature loggers); these comparisons yielded remarkably similar results, so we present only the results of the all-pairwise difference analysis. In general, the rural temperature differences were more uniform among the different rural sites, whereas the urban temperature differences exhibited more site-level variation (Supplementary Fig. S2).

**Figure 2:**
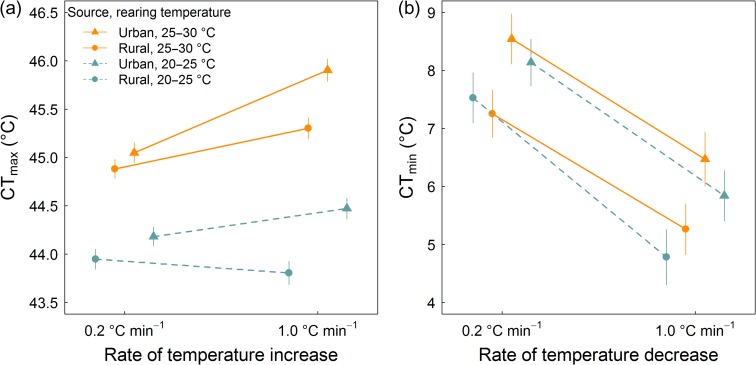
Plasticity of thermal tolerance assessed using slower and faster rates of temperature change for urban and rural acorn ant populations reared under common garden under cool and warm temperature regimes: (**a**) heat tolerance, CT_max_; and (**b**) cold tolerance, CT_min_. Predicted values (mean and standard error) from linear mixed effects models of tolerance as a function of source population (urban, rural), rearing temperature (20–25°C, 25–30°C), rate of temperature change during the thermal tolerance assay (0.2°C min^−1^, 1.0°C min^−1^) and their interaction are shown.

We found that urban populations of acorn ants are better at responding to rapid increases in temperature compared with rural acorn ant populations, that is, they achieve relatively higher CT_max_ values when environmental temperature is increased quickly compared with rural populations (Fig. 2a). In support, our statistical models revealed a significant interaction between the rate of temperature increase and source population such that urban ants tested using a fast rate of temperature increase during the thermal tolerance assay had the greatest CT_max_ values (Table 1). Post-hoc tests allowed us to further explore the nature of this interaction: we found that the magnitude of the difference between CT_max_ responses to fast versus slow rates of temperature increase was greater for urban populations than for rural populations. Indeed, we found no significant difference between rural population CT_max_ responses to fast versus slow rates of temperature increase (Table 2). Relatedly, we found that the population differentiation between urban and rural populations in CT_max_ was much more apparent under faster rates of temperature increase than under slower rates of temperature increase. In fact, while the difference between urban and rural population CT_max_ was statistically significant under the fast rate of temperature increase, it was marginally non-significant under the slow rate of temperature increase (Table 2).

**Table 1:** Statistical model summaries (estimates, standard errors, likelihood ratio test statistics and *P*-values) of CT_max_ and CT_min_ as a function of the rate of temperature change during the thermal tolerance trials, whether the source population was urban or rural, the temperature under which the colony was reared during the common garden experiment, and the two- and three-way interactions involving these variables. Significant *P*-values at the 0.05 level are indicated in bold

Tolerance type	Term	Estimate	SE	*χ* ^2^	*P*
CT_max_	Ramp rate	0.0932	0.166	21.4	**<0.0001**
*n*_obs_ = 528	Source population	0.615	0.176	21.7	**<0.0001**
*n*_groups_ = 30	Rearing temperature	1.44	0.175	190	**<0.0001**
	Ramp × Source	−0.343	0.225	7.58	**0.00592**
	Ramp × Rearing	−0.473	0.224	12.8	**0.000354**
	Source × Rearing	0.0341	0.245	0.164	0.686
	Ramp × Source × Rearing	−0.177	0.315	0.316	0.574
CT_min_	Ramp rate	2.74	0.452	112	**<0.0001**
*n*_obs_ = 526	Source population	1.06	0.644	7.35	**0.00669**
*n*_groups_ = 30	Rearing temperature	0.482	0.646	0.508	0.476
	Ramp × Source	−0.448	0.610	0.177	0.674
	Ramp × Rearing	−0.755	0.613	1.28	0.258
	Source × Rearing	0.149	0.906	0.368	0.544
	Ramp × Source × Rearing	0.530	0.858	0.382	0.537

Note that the baseline factor level for source population is the rural group and for the ramp rate is the fast group (1°C min^−1^).

**Table 2:** Post-hoc tests (estimates, standard errors, test statistics and *P*-values) for the significant interactions from models of CT_max_ (Table 1). Significant *P*-values at the 0.05 level are indicated in bold

Interaction	Difference	Group	Estimate	SE	*t*	*P*
Ramp rate × Source population	Rural—urban population	Fast ramp	−0.633	0.122	−5.19	**<0.0001**
	Rural—urban population	Slow ramp	−0.200	0.107	−1.88	0.0716
	Fast—slow ramp	Rural population	0.139	0.112	1.24	0.215
	Fast—slow ramp	Urban population	0.572	0.110	5.18	**<0.0001**
Ramp rate × Rearing temperature	Cold—warm temperature	Fast ramp	−1.46	0.122	−12.0	**<0.0001**
	Cold—warm temperature	Slow ramp	−0.900	0.107	−8.45	**<0.0001**
	Fast—slow ramp	Cold temperature	0.0742	0.112	0.663	0.508
	Fast—slow ramp	Warm temperature	0.637	0.110	5.78	**<0.0001**

Similarly, we found that CT_max_ responses to fast versus slow rates of temperature increase depended on rearing temperature, as indicated by a significant interaction between the rate of temperature increase and rearing temperature (Table 1). Post-hoc tests further showed that the difference between CT_max_ responses to fast versus slow rates of temperature increase was most pronounced at the warmer rearing temperature (Table 2).

### Ramp-rate plasticity in cold tolerance and rates of environmental temperature change

In contrast to our results for CT_max_, we saw no evidence of an interaction between the rate of temperature change during the thermal tolerance assay and source population for CT_min_, although cold tolerance was generally improved for rural acorn ant populations and when cold tolerance was assessed using a faster rate of temperature change (Fig. 2b and Table 1).

The lack of interaction between source population and the rate of temperature change was consistent with the lack of interaction between source population and rate of nest site cooling. The linear mixed model of nest site temperature as a function of hour during the diurnal period of nest site cooling, source population, and their interaction did not detect a significant interaction between hour and source population (estimate and SE: 0.184 ± 0.134, *χ*^2^ = 1.84, *P* = 0.175, though as expected urban habitats were warmer on average 0.243 ± 2.70, *χ*^2^ = 3.84, *P* = 0.0499 and temperatures in both habitats decreased over the course of the afternoon −0.905 ± 0.101, *χ*^2^ = 142, *P* < 0.0001; for each model *n*_obs_ = 1316, *n*_groups_ = 9 temperature loggers and 30 days). These results indicate that the rate of temperature decrease per hour at nest sites was the same across urban and rural environments.

### Genetic correlations between fast and slow-ramped thermal tolerance

Spearman’s rank correlations between mean colony thermal tolerance assessed under slower and faster rates of temperature change were generally weak and statistically non-significant. For CT_max_, *ρ*_urban/warm_ = 0.352, *P* = 0.439; *ρ*_rural/warm_ = −0.271, *P* = 0.516; *ρ*_urban/cool_ = −0.220, *P* = 0.601; *ρ*_rural/cool_ = 0.132, *P* = 0.778; and for CT_min_, *ρ*_urban/warm_ = −0.655, *P* = 0.111; *ρ*_rural/warm_ = 0.132, *P* = 0.756; *ρ*_urban/cool_ = −0.707, *P* = 0.0501; *ρ*_rural/cool_ = −0.127, *P* = 0.786.

## Discussion

Urban development can alter the thermal landscape, in many cases leading to more rapid changes in temperature over time and space (Imhoff *et al.*, 2010). Evolutionary differentiation among urban and rural populations in terms of their mean thermal tolerance has been described in some systems (Brans *et al.*, 2017; Diamond *et al.*, 2017), and urban-driven evolutionary change in sub-lethal performance traits like growth rate, which captures the plastic response of growth across a range of rearing temperatures, has likewise been demonstrated (McLean *et al.*, 2005; Tüzün *et al.*, 2017). But whether the plastic response of thermal tolerance to different rates of temperature change evolves in urban environments is unclear. In this study, we examined how plasticity in thermal tolerance across faster and slower rates of temperature change (the “ramp rate”) evolves in response to the more rapid rate of temperature change experienced in cities. Using a common garden experimental design with acorn ants from urban and rural populations, we found that, relative to rural population ants, urban population ants exhibited greater heat tolerance under the fast rate of temperature change, and this result was correlated with both faster rates of diurnal temperature rise in urban acorn ant nest sites and more rapid spatial changes in temperature across urban foraging areas. These results underscore the importance of linking ramp rate plasticity in thermal tolerance traits with ecologically relevant variation in the rate of temperature change in the field, and further demonstrate the need to examine not only evolutionary differentiation in mean trait values, but also trait plasticity.

The sensitivity of thermal tolerances to the nature of temperature stress—whether static or dynamic, and in the latter case, whether the temperature change is faster or slower—is well established for a number of ectothermic species (Allen *et al.*, 2016; Castañeda *et al.*, 2015; Chown *et al.*, 2009; Moyano *et al.*, 2017; Overgaard *et al.*, 2012; Terblanche *et al.*, 2011). For the dynamic assessment of thermal tolerance, where a temperature stress is applied incrementally over time until the loss of muscular coordination (the critical temperatures, CT_max_ and CT_min_) or until death (the lethal thermal limits), the relative merits of faster or slower rates of temperature change have been debated for some time (Terblanche *et al.*, 2011). The debate is also far from an academic one: the differences in tolerance estimates using faster or slower rates of temperature change can be substantial, on the order of several degrees Celsius. Faster rates of temperature change typically lead to upward bias in the magnitude of tolerance, either improved heat tolerance or improved cold tolerance, potentially as a consequence of minimizing confounding effects including starvation and desiccation (Chown *et al.*, 2009; Rezende *et al.*, 2011; Santos *et al.*, 2011, 2012). The tradeoff with faster rates of temperature change mitigating potential confounding variables is that they have been criticized as having less ecological relevance. By contrast, while slower rates of temperature change may be more ecologically relevant, they may also be confounded non-temperature aspects of physiological stress (but see Overgaard *et al.*, 2012). A major assumption here is that slow rates of temperature change are the most ecologically relevant—for decades the most commonly used ramp rate was ~0.3°C min^−1^ as a compromise between fast and slow rates of temperature change, though positioned more toward the slower rate of temperature change (reviewed in Allen *et al.*, 2016). Recent syntheses show that while slow rates of temperature change are appropriate in many scenarios, there is considerable variation in the rate of environmental temperature change in nature (Terblanche *et al.*, 2011). Below we explore the nature of environmental temperature variation and challenge the assumption of the ecological relevance of slow rates of temperature change, particularly in urbanized landscapes.

First, however, our results provide clear evidence of the substantial influence of ramp rate on tolerance estimates, irrespective of how well ramp rates used during laboratory assays match environmental rates of temperature change. We generally find support for upward bias in the estimates, such that faster rates of temperature change yield improved heat tolerance (higher CT_max_ values) and cold tolerance (lower CT_min_ values). The effect of ramp rate was greater for CT_min_ than for CT_max_ (Table 1), though this is consistent with other findings from ectothermic species which show more limited plasticity in heat tolerance under differences in mean rearing temperature (Gunderson and Stillman, 2015; Sørensen *et al.*, 2016). Additionally, we found contrasting roles for the interactive effects of rearing temperature and ramp rate on CT_min_ and CT_max_. While we found that the effect of ramp rate was independent of rearing temperature for CT_min_, we found that the relationship between CT_max_ and the rate of temperature increase depended on the acclimation temperature (Table 1). Specifically, the increase in CT_max_ under faster rates of temperature change was greater in the warmer acclimation temperature (Fig. 2a and Table 2). Although few tests have been performed, the interactive effect of ramp rate and acclimation temperature on thermal tolerance is supported by work in other arthropod systems (Allen *et al.*, 2016; Castañeda *et al.*, 2015). Indeed, Allen *et al.* 2016, further show that heat tolerance response to different rates of temperature change varies among species, demonstrating that ramp rate plasticity in thermal tolerance can evolve. This finding suggests ramp rate plasticity may evolve over long timescales, but leaves open the question of whether ramp rate plasticity in thermal tolerance can evolve rapidly over contemporary timescales.

Cities provide natural laboratories to explore the rapid evolution of tolerance plasticity, specifically how urban-driven environmental differences in the rate of temperature change among populations shape the tolerance response to different rates of temperature change during the tolerance assessment. Many cities exhibit substantial heat island effects wherein urban environments are warmer (by several °C) than nearby undeveloped areas owing to high levels of impervious surfaces like roads, sidewalks and buildings that absorb and trap heat, and diminished vegetation cover which can mitigate the effects of temperature rise (Imhoff *et al.*, 2010). For the acorn ants used in our study, we found faster rates of diurnal temperature rise at urban nest sites during peak diurnal activity periods in the morning and early afternoon (Fig. 1a). We also found at least the potential for acorn ants to experience more rapid rates of spatial temperature change across urban foraging areas that had sparser canopy cover and thus increased amounts of warmer, light gap microclimates (Fig. 1b). These more rapid spatio-temporal environmental temperature increases in urban habitats were associated with greater plasticity in urban acorn ant heat tolerance across slower to faster rates of temperature change. Specifically, the urban population ants exhibited significantly greater increases in heat tolerance from the slower to faster rate of thermal tolerance assessment compared with rural population ants (Fig. 2a and Table 1). Together, these results provide evidence for evolved plasticity among urban and rural populations in the response of their heat tolerance to different rates of temperature change, and are consistent with the direction of environmental temperature variation (more rapid spatio-temporal variation in urban habitats).

Notably, rural populations of acorn ants are already accustomed to relatively abrupt temperature changes, such as moving from the nest environment which shields ants against direct solar radiation to the more exposed foraging environment, and between light gaps in the canopy cover; however, these temperature changes appear to be exaggerated in urban environments. We found that the magnitude of temperature change across the foraging area (estimated by the difference in the peak of the kernel density distributions of temperature) in rural habitats ranged from 0.361 to 0.586°C, and from 0.752 to 1.12°C in urban habitats. But what do these temperature differences mean to a foraging acorn ant? Acorn ant workers are small in body size (~2 mm), but a long-distance foraging trip of 1 m would only take 1.11–2.08 min depending on ambient thermal conditions (at 26°C, a common foraging temperature in both rural and urban habitats, acorn ant workers travel on average 8 mm s^−1^, and at 36°C, a warm-day foraging temperature, acorn ant workers travel on average 15 mm s^−1^, Maclean *et al.*, 2017). As a consequence, foraging acorn ants experience quite rapid changes in temperature across space, particularly in cities, such that our fast rate of temperature change during the thermal tolerance assay, 1°C min^−1^, appears to reflect the temperature changes experienced by urban acorn ant foragers. By contrast, the 0.2°C min^−1^ rate of temperature change during the thermal tolerance assay appears to better reflect the temperature changes experienced by rural acorn ant foragers. The nest site temperature changes were more subdued than foraging area temperature changes, but were nonetheless twice as fast during peak activity hours (mid-morning to early afternoon) in urban habitats compared with rural habitats, i.e. 2.2°C versus 1.2°C h^−1^.

So far, we have established that temperature change in urban habitats is more rapid at nest sites and foraging areas of acorn ants, that foragers have sufficiently fast running speeds to at least potentially experience the large magnitude of temperature changes across the foraging area, and that plasticity in heat tolerance is correlated with these rapid temperature changes in urban habitats (Figs 1 and 2; Table 1; Maclean *et al.* 2017). But is the magnitude of plasticity in heat tolerance under different rates of temperature change ecologically meaningful? Under the warm laboratory temperature regime (30°C daytime, 25°C nighttime), the increase in heat tolerance from slower to faster rates to temperature increase was on the order of 1°C, a value comparable to other ectothermic species (fruit flies show an increase of 1°C and Argentine ants an increase of several°C from a rate of temperature increase of 0.1–0.5 min^−1^, Chown *et al.*, 2009), and of sufficient magnitude to alter fitness outcomes in ants (Penick *et al.*, 2017). It is unclear whether this greater plasticity in urban acorn ant responses to faster rates of temperature increase is adaptive; however, it is plausible the shift is adaptive, as an increased ability to tolerate more rapid temperature rise could allow workers to sustain foraging in the highly heterogeneous foraging areas in urban habitats. For many ant species, foraging rate is related to colony growth which subsequently allows for the production of sexual reproductives (alates) when colonies become sufficiently large (reviewed in Shik, 2008).

Another open question is the extent to which tolerance responses to slower and faster rates of temperature change are genetically correlated. The evidence so far, and mostly from studies on the genetic architecture of thermal tolerance in *Drosophila* systems, indicates that in some cases, basal tolerance (akin to the fast-ramp tolerance in our study, assuming this is a sufficiently fast rate of temperature change to preclude an acclimatory response and which therefore captures the intrinsic genetic contribution to tolerance) and induced tolerance (akin to the slow-ramp tolerance in our study, assuming this is a sufficiently slow rate of temperature change to allow for acclimation) responses are positively correlated (Hangartner and Hoffmann, 2016; van Heerwaarden and Sgrò, 2013). Though in other cases, basal and induced heat tolerance responses are uncorrelated, suggesting at least some degree of independence in the genetic architecture of these components of tolerance (Blackburn *et al.*, 2014; Mitchell and Hoffmann, 2010). We have neither a selection experiment nor a quantitative genetics breeding design as used in these *Drosophila* studies. However, we were able to explore the potential for correlation among faster- and slower-ramped tolerance responses (including both heat and cold tolerance) using our split-colony experimental design which provides an estimate of genetic correlation between fast- and slow-ramped traits. We computed colony mean fast- and slow-ramped thermal tolerance within each combination of urban versus rural populations and cool versus warm temperature treatments. We performed separate correlation analyses for heat and cold tolerance. Our analyses revealed little evidence of a consistent positive trend between tolerances assessed under slower and faster rates of temperature change across any of these groups, suggesting a weak or nonexistent genetic correlation. However, caution must be exercised here given the limitations on sample size and experimental design to test the genetic architecture of tolerance to different rates of temperature change.

Beyond linking evolved plasticity in heat tolerance under different rates of warming with environmental variation in rates of warming and exploring the genetic architecture of different components of heat tolerance, there are other important implications of our findings concerning the nature of population differentiation in heat tolerance. Differentiation between urban and rural populations appeared to be much greater in the intrinsic genetic component of heat tolerance (captured by the fast temperature ramp) rather than the induced acclimatory component of heat tolerance (captured by the slow temperature ramp) (Tables 1 and 2). Indeed, the evolutionary differentiation in heat tolerance between urban and rural populations was only detectable (statistically significant) when using the fast temperature ramp; differentiation was much weaker and marginally non-significant when using the slow temperature ramp (Table 2). The corollary of this finding is that although both fast and slow ramped heat tolerances suggest the evolution of improved heat tolerance in urban populations, the interpretation of the magnitude of the population differentiation would be quite different if only the fast ramp or the slow ramp assessment of heat tolerance was used. Further, this pattern appears to be largely driven by urban population responses to differences in the rate of temperature change, as the heat tolerance of the rural population was insensitive to whether fast or slow rates of temperature change were used (Table 2; though there was a trend for improved heat tolerance of the rural population from the slow to fast ramp under the warm rearing temperature treatment, Fig. 2a). This diminished sensitivity of the rural population to variation in the rate of temperature change is perhaps unsurprising given the relative homogeneity of rural thermal environments over time and space (Fig. 1). In any case, these results broadly support the growing importance of considering multiple components of thermal tolerance. For example, in *Drosophila*, geographic clines in thermal tolerance can be concluded to be present or absent based solely on which type of thermal tolerance assay is used (Sgrò *et al.*, 2010); similar to our results, Castañeda and colleagues (2015) detected significant population differentiation in CT_max_ across a latitudinal cline only when using intrinsic measures of heat tolerance, but found no cline when using ramping measures of heat tolerance. Although this would seem to suggest a preference for using intrinsic measures of heat tolerance, other studies have found tolerance to be improved under slow rates of temperature change (Sørensen *et al.*, 2013; and similar exceptions exist for cold tolerance, see Powell and Bale, 2004). As populations can diverge in some components of tolerance but not others, and the nature of this divergence depends on the specific study system, the choice of tolerance assay should be considered carefully when undertaking comparative studies of thermal tolerance.

Although we found evidence of plasticity in heat tolerance of acorn ants tested under slower and faster rates of temperature increase, and that the magnitude of this plasticity differed among urban and rural populations, we found no evidence of plasticity in cold tolerance under slower and faster rates of temperature decrease. However, we did find evidence of evolved losses in mean cold tolerance among urban populations—it is unclear why cold tolerance is lost, and in part appears to reflect a correlated response with changes in heat tolerance (Diamond *et al.*, 2017).

The explanation for no differences among urban and rural populations in their ramp-rate plasticity in cold tolerance is perhaps simpler: we did not observe significant differences in rates of cooling at the nest site (Fig. 1a). One caution with this interpretation is that our study was much more focused on growing season responses (ant colonies were reared in common garden under growing season temperature regimes), and the lack of plasticity in cold tolerance could simply reflect the fact that ants were not tested for cold tolerance while being reared under overwintering conditions. However, we might expect that the lack of ramp-rate plasticity in cold tolerance could be reinforced by the insulating effects of snow cover over winter (Williams *et al.*, 2015). Indeed, snow cover may buffer acorn ants against differences in rates of air temperature change across urban and rural habitats.

One important limitation of our study is the lack of information on how acorn ant foragers use the foraging area in rural and urban habitats. Although the rate of temperature increase during peak activity periods at nest sites is clearly greater in urban habitats (though small modifications in temperature can be achieved by moving from the top to bottom of the nest, see Karlik *et al.* (2016)), it is possible that foragers may avoid extreme temperatures in the foraging environment by excluding these microclimates from their foraging routes. Even as ant foragers move through warmer sections of the foraging area, the difference between air temperature and body temperature (Paaijmans *et al.*, 2013; Sunday *et al.*, 2014) may also afford some degree of thermal buffering against extreme temperatures in urban environments. Nonetheless, the association between faster rates of temperature change in urban environments and the greater plasticity in heat tolerance of urban acorn ant populations suggests that acorn ants are likely incapable of completely avoiding these more rapidly changing urban thermal environments.

In this study, we have further moved the debate on the use of faster versus slower rates of temperature change during thermal tolerance assays from the laboratory to the field. Specifically, we have linked microclimatic variation in the rate of temperature increase experienced by urban acorn ants to greater evolved plasticity in urban populations of acorn ants to respond, via improved heat tolerance, to these rapid temperature changes. Forecasts of species responses to global change may therefore benefit from a more fine-scale consideration of how thermal environments vary over space and time, and how these environmental differences in the rate of temperature change shape the evolution of thermal tolerance plasticity.

## Supplementary Material

Supplementary Data
