# Supplementary material for: Evolution of plasticity in the city: urban acorn ants can better tolerate more rapid increases in environmental temperature
Source: Conserv Physiol. 2018 Jun 14;6(1):coy030. doi: 10.1093/conphys/coy030 (PMC6007456; doi:10.1093/conphys/coy030)
Supplement: Supplementary Data [file coy030_diamond_cons_phys_supplementary_data.pdf]

Supplementary data

Supplementary Table 1. Geographic coordinates and habitat designation (urban or rural) for the collection of acorn ant colonies for the common garden experiment and monitoring of environmental temperatures at nest sites and foraging areas.

Site usage	Latitude	Longitude	Habitat designation
Common garden experiment	41.509813	-81.614191	urban
	41.508968	-81.613669	urban
	41.505083	-81.609003	urban
	41.508556	-81.61302	urban
	41.6088	-81.312686	rural
	41.49489	-81.429446	rural
	41.496242	-81.42928	rural
Nest site temperature monitoring only	41.504005	-81.607673	urban
	41.493409	-81.426348	rural
Foraging area and nest site temperature monitoring	41.527755	-81.573808	urban
	41.527622	-81.573776	urban
	41.527493	-81.573748	urban
	41.504188	-81.607803	urban
	41.493486	-81.42647	rural
	41.493437	-81.426473	rural
	41.493364	-81.42647	rural

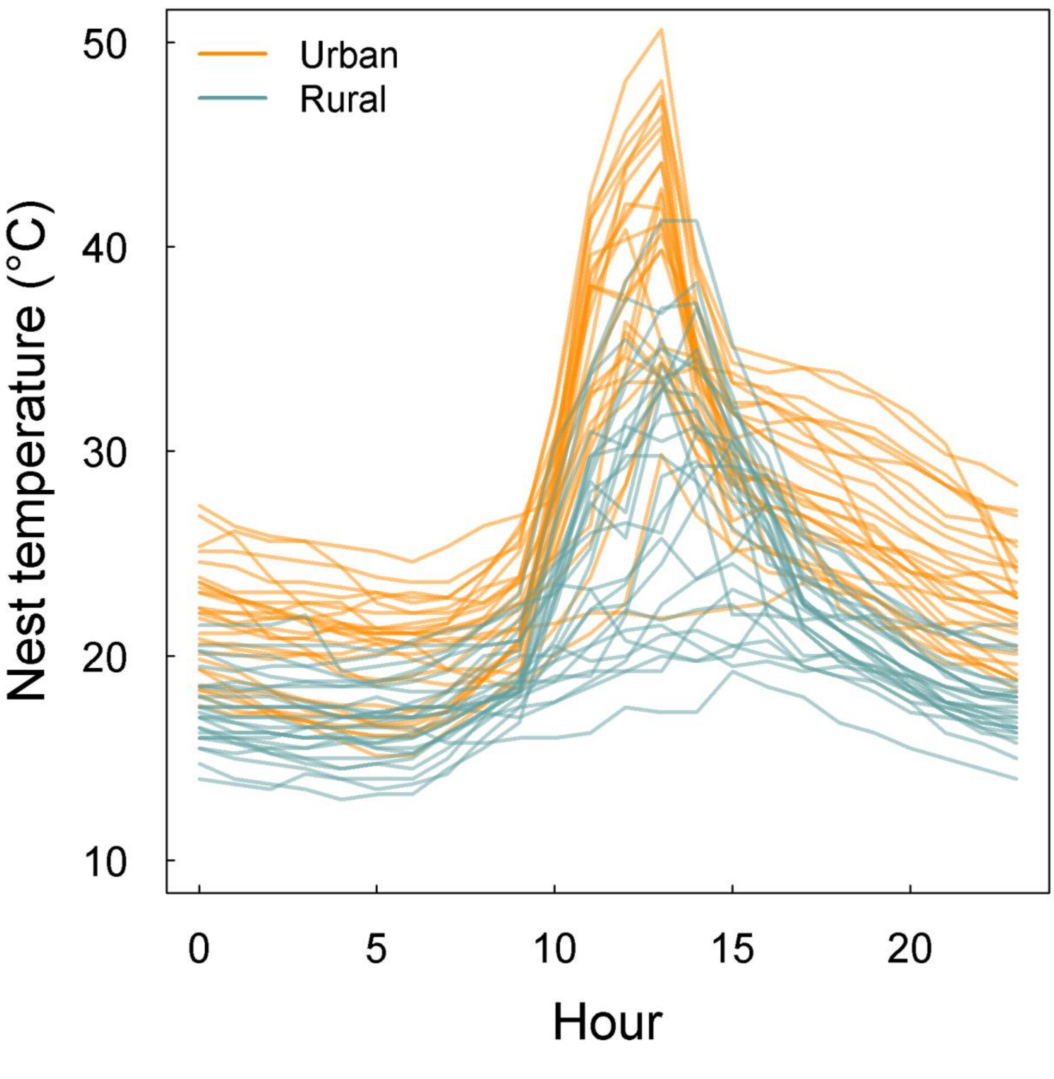

Supplementary Figure 1. Diurnal temperature profiles of acorn ant nest sites for a single rural and single urban habitat from June to July.

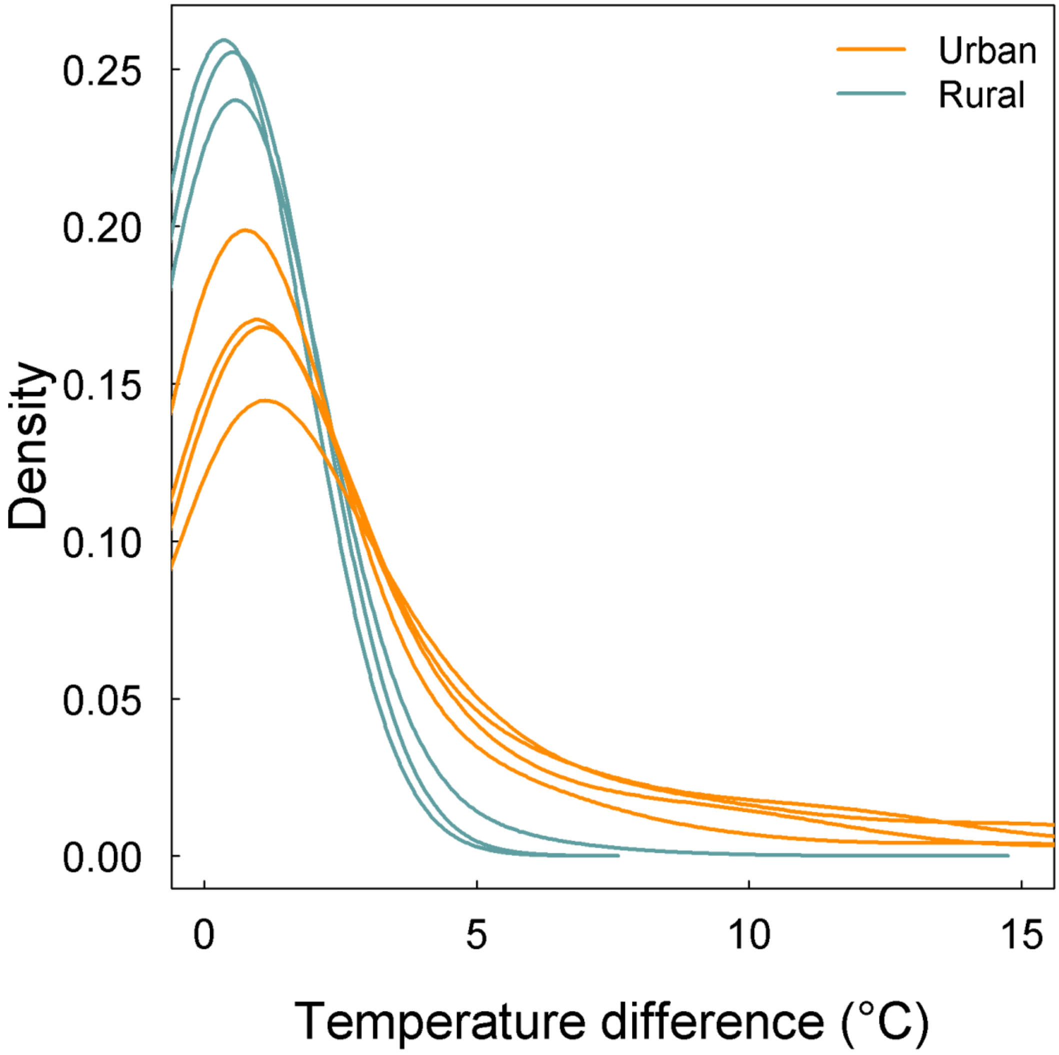

Supplementary Figure 2. Kernel density distributions (smoothed histograms) of the hourly pairwise differences (during the peak foraging interval, 9 a.m. to 1 p.m.) among foraging area temperatures for urban and rural habitats during peak activity season (June-July). Each monitoring site is presented separately.
